# Impact of AlF_3_-CaB_4_O_7_ Doping on Terahertz Dielectric Properties and Feasibility of Low/Ultra-Low Temperature Co-Fired Ceramics

**DOI:** 10.3390/ma18184272

**Published:** 2025-09-12

**Authors:** Beata Synkiewicz-Musialska, Dorota Szwagierczak

**Affiliations:** Łukasiewicz Research Network-Institute of Microelectronics and Photonics, ul. Zabłocie 39, 30-701 Kraków, Poland

**Keywords:** low/ultra-low temperature co-fired ceramics, terahertz dielectric properties, low dielectric permittivity, willemite, copper borate, lithium borate, lithium tungstate

## Abstract

Modification of the composition by doping is an effective way to develop new substrate materials for 5G/6G communication systems. This paper aims to study the impact of AlF_3_-CaB_4_O_7_ doping on dielectric properties at very high frequencies, sintering temperature, microstructure, and feasibility in LTCC/ULTCC (low/ultra-low temperature cofired ceramics) technology of four low dielectric permittivity materials based on CuB_2_O_4_, Zn_2_SiO_4_, LiBO_2_, and Li_2_WO_4_. Sintering behavior, microstructure, elemental and phase composition, and dielectric properties in the terahertz range were characterized using a heating microscope, SEM, EDS, XRD methods, and time domain spectroscopy. The developed ceramics exhibit excellent dielectric behavior at terahertz frequencies and are feasible in ULTCC or LTCC technology. These properties make them good candidates for substrates in 5G/6G communication systems.

## 1. Introduction

New substrate materials with a low and temperature-stable dielectric permittivity and low dielectric loss in the terahertz frequency range and with sufficiently low sintering temperatures to be feasible in LTCC/ULTCC technology (low/ultra-low temperature cofired ceramics) are pivotal for the development of advanced 5G and 6G communication systems [1,2]. Since the signal propagation delay is proportional to the square root of the dielectric permittivity ε_r_ of the substrate, the minimization of its value is crucial for the very high speed of signal transmission. Low dielectric loss (low loss tangent, high quality factor Qxf) reduces energy dissipated as heat and improves frequency selectivity. The temperature stability of the substrate’s dielectric properties is important for maintaining a stable operational frequency of an electronic device, regardless of temperature fluctuations [3,4,5,6,7]. Two strategies are used to adjust the temperature coefficient of the resonant frequency of a substrate material to the desired level close to zero. One approach is the introduction of an additive with a positive τ_f_ value in order to compensate for the negative τ_f_ value of the basic ceramic component. There are several materials used as such compensators: TiO_2_, Li_2_TiO_3_, CaTiO_3_, Li_2_TiGeO_5_, Li_2_TiSiO_5_, TiB, SrNb_2_V_2_O_11_ [4,5,6]. The second approach to lowering the temperature coefficient of the resonant frequency is appropriate doping, which can lead to the formation of solid solutions [7], related to the modification of the cell parameters, bond length, and internal stress in the crystal lattice.

LTCC/ULTCC technology promotes better miniaturization and integration of passive components. It also reduces energy and fabrication costs owing to a simple and flexible procedure, leading to the final cofiring of a multilayer module consisting of several functional layers. LTCC ceramics should have a sintering temperature in the range of 850–1000 °C, which enables cofiring with commercial thick film conductor Ag pastes (melting point of silver is about 960 °C) or AgPd pastes with a firing temperature below 1000 °C. ULTCC ceramics require much lower firing temperatures (550–700 °C), adjusted to cheap Ag pastes or Al pastes (melting point of aluminum is about 660 °C).

Using appropriate additives is often required to attain the desired properties and thermal treatment conditions. These additives should ensure both lowering or stabilizing the sintering process and preserving good dielectric properties of the main ceramic component of the LTCC/ULTCC substrate. Among the effectively used additives for low dielectric permittivity LTCC ceramics are some borates or borate glasses [8,9,10], as well as some fluorides, such as LiF, MgF_2_, CaF_2_, and BaF_2_ [11,12,13,14,15,16,17,18,19].

All materials that are the subject of the present study show low intrinsic dielectric permittivities (4.5–6.5). Among these materials, the best known is Zn_2_SiO_4_ [14,15,20,21,22,23,24]. Its high sintering temperature, exceeding 1300 °C, can be successfully lowered using proper additives. Recently, Yang et al. investigated nonstoichiometric willemite Zn_1.8_SiO_3.8_ with the sintering temperature effectively reduced to 950 °C by LiF [14] or MgF_2_ [15] addition. These authors stated that LiF-based liquid phase filled the pores and facilitated the sintering process. It was revealed that part of Li^+^ and F^−^ ions entered the crystal lattice of Zn_2_SiO_4_, while the remaining LiF participated in the formation of a Si-rich amorphous phase [14]. The best densification degree and optimal microwave dielectric properties were reported for 2 wt% LiF content—ε_r_ of 6.3, Qxf of 64,200 GHz, and τ_f_ of −21.1 ppm/°C [14]. The addition of an appropriate amount of MgF_2_ (≤2 wt%) entailed the modification of the lattice energy and defect structure, which resulted in a reduction of both intrinsic and extrinsic losses. This enabled obtaining the ceramic with very good dielectric properties—ε_r_ of 6.45, Qxf of 127,500 GHz, and τ_f_ of −25.1 ppm/°C [15]. Chaware et al. obtained Zn_2_SiO_4_-based ceramics with a sintering temperature lowered to 950 °C owing to the addition of zinc borate Zn_3_B_2_O_6_. Zn_2_SiO_4_-Zn_3_B_2_O_6_ composite had good microwave dielectric properties-ε_r_ of 6.1 and quality factor of 94,300 GHz [20]. Kim et al. described a nonstoichiometric Zn_1.8_SiO_3.8_ ceramic with the addition of 20 mol% B_2_O_3_, which showed very good microwave properties after sintering at 900 °C: ε_r_ = 5.7, Qxf = 53,000 GHz, and ε_r_ = −16 ppm/°C [21]. Du et al. developed a 0.95 Zn_2_SiO_4_-0.05 CaTiO_3_ composite with the addition of 4 wt.% Li_2_CO_3_-H_3_BO_3_ with a sintering temperature of 950 °C, a low dielectric permittivity of 7.1, a Qxf of 26,300 GHz, a near-zero ε_r_ of −4.5 ppm/°C, and good chemical compatibility with silver [22]. Synkiewicz et al. and Szwagierczak et al. effectively lowered the sintering temperature of the willemite ceramic to 900–980 °C by doping with mixtures of Li_2_TiO_3_, MgTiO_3_, AlF_3_, and CaB_4_O_7_ [23] and Zn_4_B_6_O_13_ [24]. The obtained ceramics showed a low dielectric permittivity of 5.8–6.8 at 1 THz [23,24].

Other investigated materials, CuB_2_O_4_ [13,25,26,27,28], LiBO_2_ [29,30,31,32], and Li_2_WO_4_ [33,34,35,36,37], which have sintering temperatures suitable for LTCC/ULTCC technology, are known from other applications, such as thermoluminescent, magnetooptic, piezoelectric, and electrocatalytic materials, although they have scarcely been investigated as substrates for microwave or terahertz circuits. In our previous works, we studied ceramics based on copper borates and successfully used these materials for the fabrication of green tapes and LTCC structures [13,28]. The developed CuB_2_O_4_-Cu_3_B_2_O_6_ composites doped with Li-containing additives—Li_2_WO_4_, LiBO_2_, and Li_2_CO_3_—were sintered at 860–880 °C and exhibited a low dielectric permittivity of 5.4–5.7 and a low loss tangent of 0.008–0.01 at 1 THz [13]. CuB_2_O_4-_Cu_3_B_2_O_6_ composites doped with 5% CuBi_2_O_4_ had optimal sintering temperatures of 900–920 °C and low dielectric permittivities of 5.8–6.1 in the 0.14–0.7 THz range [28]. Yin et al. developed a single-phase LiBO_2_ ceramic sintered at 640 °C with a low dielectric permittivity of 5.3, a moderate quality factor of 18,200 GHz at 16.3 GHz, a temperature coefficient of resonant frequency of −66.2 ppm/°C, a thermal expansion coefficient of 25.4 ppm/°C, and good compatibility with Ag electrodes [31]. The good microwave properties of Li_2_WO_4_ ceramics—*ε_r_* of 5.5, *Q* × *f* of 62,000 GHz, and *τ_f_* of −146 ppm/°C at 15.7 GHz were described by Zhou et al. [33]. Sasidharanpillai et al. fabricated Li_2_WO_4_ green tapes using environmentally friendly organic additives and obtained substrates sintered at 650 °C with excellent microwave dielectric properties-a low dielectric permittivity of 5.4, a very low dielectric loss of 9.21 × 10^−5^ for 5 GHz, and co-sinterability with Ag electrodes [34]. Szwagierczak et al. developed ULTCC substrates based on Li_2_WO_4_ with AlF_3_–CaB_4_O_7_ and CuBi_2_O_4_ additives. These substrates exhibited an ultra-low sintering temperature of 590–630 °C, compatibility with Ag conductors, low dielectric permittivity of 5.0–5.8 in a broad range of 0.2–2 THz, and low loss tangent of 0.008–0.01 at 1 THz [35].

The search for new LTCC substrate materials for 5G/6G communications requires the implementation of both the new basic materials with excellent high-frequency dielectric properties and the new additives lowering the sintering temperature. This research presents the impact of a new additive, the mixture of fluoride AlF_3_ and borate CaB_4_O_7_ applied earlier only in our previous works, on the sintering behavior, microstructure, and terahertz dielectric properties of four low dielectric permittivity ceramic materials: willemite Zn_2_SiO_4_, copper borate CuB_2_O_4_, lithium borate LiBO_2_, and lithium tungstate Li_2_WO_4_.

The purpose of this study is to elaborate on the composition and fabrication procedures of four ceramics doped with AlF_3_-CaB_4_O_7_ and to compare the properties of test green tapes and LTCC/ULTCC substrates with a special focus on their terahertz dielectric properties.

## 2. Materials and Methods

Ceramic materials were synthesized via the solid-state reaction method performed at 600 °C for Li_2_WO_4_, 700 °C for CuB_2_O_4_, 740 °C for LiBO_2_, and 1150 °C for Zn_2_SiO_4_, as described in our previous works [13,23,28,32,35]. Each of the synthesized powders was ball-milled (Pulverisette 5, Fritsch, Germany) for 8 h in isopropyl alcohol and dried. Then 4–5 wt.% of the dopant, being the eutectic mixture of AlF_3_ and CaB_4_O_7_ in a 1:1 molar ratio, was added to each powder and subsequently ball-milled for 8 h. The applied dopant has an optimal composition with the lowest melting point established based on a systematic study using a heating microscope for various contents of AlF_3_ (20–80 mol%) in the AlF_3_-CaB_4_O_7_ mixture. The amount of the introduced dopant was optimized at a 4–5 wt% level based on the studies performed for the range of 2–10 wt%.

The ceramic powders were used for uniaxial pressing and sintering of disc-shaped samples or for the preparation of multilayer substrates in an LTCC/ULCC process comprising tape casting (TTC-1200, Mistler, Morrisville, PA, USA), screen printing of conductive layers (Microtec MT-320TVC, Urayasu, Japan), isostatic lamination (IL-4008PC, Pacific Trinetics Corporation, Fremont, CA, USA), and co-sintering of ceramic tapes with metallic thick films. Three commercial pastes were used for screen printing test conductor patterns: ESL Ag9916, ESL AgPd9638, and DuPont Ag7713. A digital optical microscope (KH-7700, Hirox, Tokyo, Japan) was used to evaluate the quality of screen-printed patterns.

The sintering behavior, microstructure, and phase composition were characterized using heating microscope observations (Leitz, Wetzlar, Germany), SEM and EDS analysis (FEI Nova Nano SEM 200 with EDAX Genesis EDS system, Hillsboro, OR, USA, and Quattro S Thermo Fisher Scientific, Loughborough, UK), and XRD analysis (Empyrean, PANalytical, Almelo, The Netherlands). Dielectric properties in a broad frequency range of 0.2–3 THz were investigated using the time domain spectroscopy (TDS) method (TPS Spectra 3000, Teraview, Cambridge, UK) [32]. The main source of measurement error was the variation of the sample’s thickness. The values of dielectric permittivity and loss tangent derived from the TDS measurements were determined with an uncertainty level of 1–2%.

## 3. Results and Discussion

One of the preconditions for the feasibility of an LTCC/ULTCC substrate is adjusting the sintering temperature of the material to the requirements of this technology.

**Figure 1:**
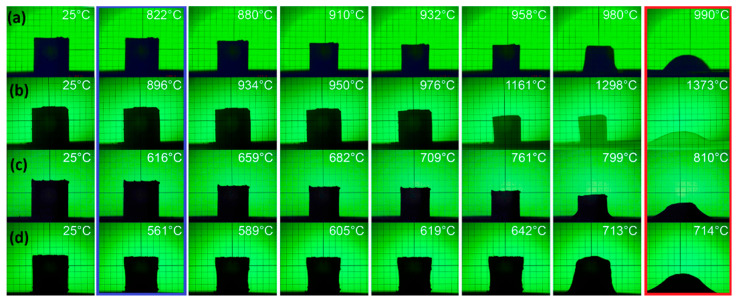
Images from a heating microscope for ceramics doped with AlF_3_-CaB_4_O_7_: (**a**) CuB_2_O_4_; (**b**) Zn_2_SiO_4_; (**c**) LiBO_2_; (**d**) Li_2_WO_4_. Blue frame: temperatures of the start of shrinking, red frame: melting temperatures.

Figure 1 presents selected images from a heating microscope, which reflect how the shape and dimensions of the investigated samples changed during heating in the range from room temperature to their melting points. Table 1 presents a comparison of characteristic temperatures derived from the heating microscope observations for each of the undoped and doped ceramics—the temperature of the start of shrinking, the sintering temperature range, and the melting temperature.

For all materials, lowering of the onset of shrinking and overall improvement of the densification process and microstructure uniformity were observed as a result of using AlF_3_-CaB_4_O_7_ as an additive. From Figure 1 and Table 1, it follows that the start of shrinking was observed at 561 °C for Li_2_WO_4_, 616 °C for LiBO_2_, 822 °C for doped CuB_2_O_4_, and 896 °C for doped Zn_2_SiO_4_. The optimal sintering ranges were established as 900–940 °C for doped CuB_2_O_4_, 950–980 °C for doped Zn_2_SiO_4_, 650–680 °C for doped LiBO_2_, and 590–620 °C for doped Li_2_WO_4_. LiBO_2_ and Li_2_WO_4_ can be classified as ULTCC materials, while doped CuB_2_O_4_ and doped Zn_2_SiO_4_ can be useful as LTCC materials.

The AlF_3_-CaB_4_O_7_ additive promotes liquid phase-assisted sintering of the investigated materials at temperatures lower than those for pure ceramics. Consequently, doping with AlF_3_-CaB_4_O_7_ results in a decrease in the sintering temperature of each of the pure ceramics. This effect is very strong for Zn_2_SiO_4_ (360 °C) and moderate or small for CuB_2_O_4_ (60 °C), LiBO_2_ (40 °C), and Li_2_WO_4_ (40 °C).

**Figure 2:**
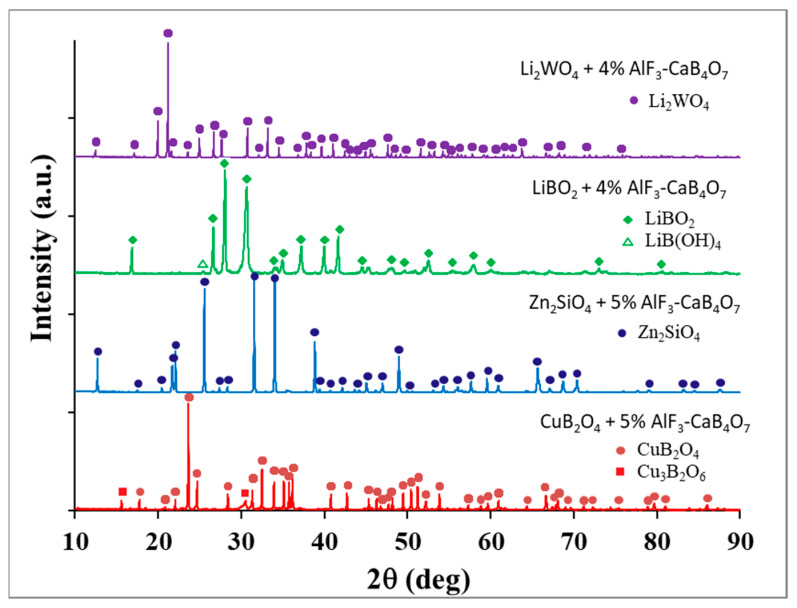
XRD patterns for CuB_2_O_4_, Zn_2_SiO_4_; LiBO_2_ and Li_2_WO_4_ ceramics doped with AlF_3_-CaB_4_O_7_.

Based on the XRD results, it was stated that the phase compositions of the doped and undoped ceramics were similar for each of the investigated materials. Figure 2 compares the XRD patterns for CuB_2_O_4_, Zn_2_SiO_4_, LiBO_2_, and Li_2_WO_4_ ceramics doped with AlF_3_-CaB_4_O_7_. The detected crystalline phases are CuB_2_O_4_ (tetragonal system, I-42d space group—Ref. code 98-002-0273), Zn_2_SiO_4_ (hexagonal system, R-3 space group—Ref. code 98-000-2425), LiBO_2_ (monoclinic system, space group P121/c1—Ref. code 98-020-0891), and Li_2_WO_4_ (hexagonal system, space group R-3—Ref. code 98-001-4196).

Generally, no additional crystalline phases originating from the introduction of the dopant were found on the XRD patterns. The only secondary crystalline phases revealed in small amounts were another copper borate Cu_3_B_2_O_6_ (triclinic system, P-1 space group, Ref. code 98-003-5201) for CuB_2_O_4_ and lithium hydrate LiB(OH)_4_ (orthorhombic system, space group P bca—Ref. code 98-002-7316), which appeared for doped LiBO_2_ due to the susceptibility of this compound to hydration [29]. Furthermore, the XRD analysis showed a significant amount of an amorphous phase in the samples, at the level of 8–12%. The lack of crystalline phases based on the introduced AlF_3_-CaB_4_O_7_ additive in the XRD diffractograms implies that the dopant partly enters the crystal lattice of the main ceramic components or forms an amorphous phase at grain boundaries.

**Figure 3:**
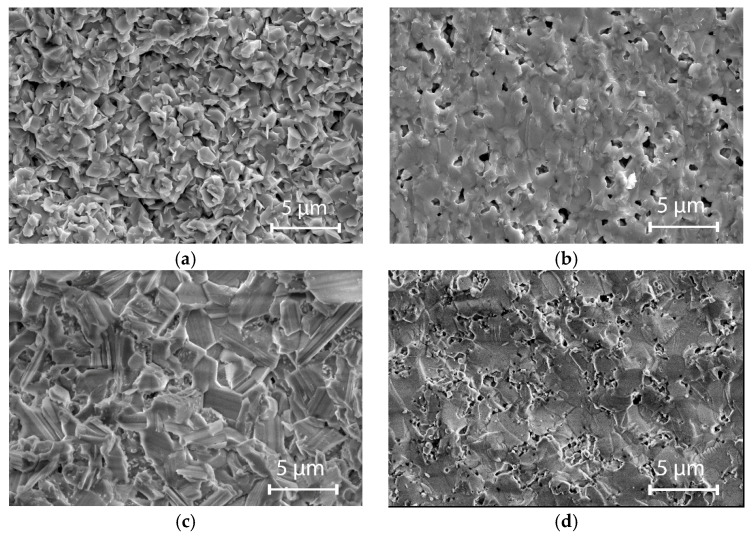
SEM images of fractured cross-sections for ceramics doped with AlF_3_-CaB_4_O_7_: (**a**) CuB_2_O_4_ sintered at 900 °C; (**b**) Zn_2_SiO_4_ sintered at 960 °C; (**c**) LiBO_2_ sintered at 660 °C; (**d**) Li_2_WO_4_ sintered at 600 °C.

Figure 3 presents SEM images of the fractured cross-sections of the doped ceramics. All ceramics are well sintered and fine-grained. The most uniform microstructure, with small grains with sizes in the 0.5–3 μm range, is observed for CuB_2_O_4_ ceramics sintered at 900 °C (Figure 3a). SEM images of doped willemite ceramics sintered at 950 °C (Figure 3b) and doped Li_2_WO_4_ sintered at 600 °C (Figure 3d) reveal grain growth, poorly visible grain boundaries, and small closed porosity. The microstructure of doped LiBO_2_ ceramic is dense with uniform grain sizes ranging from 1 to 5 μm (Figure 3c). The largest grain sizes were found for doped Li_2_WO_4_ ceramics (1–6 μm) (Figure 3d). The microstructures of the undoped [23,28,32,35] and doped materials do not differ significantly. However, for the doped ceramics, a good densification degree with a relative density exceeding 95% can be attained at lower temperatures, and consequently, optimal sintering temperatures are lower (Table 1).

**Figure 4:**
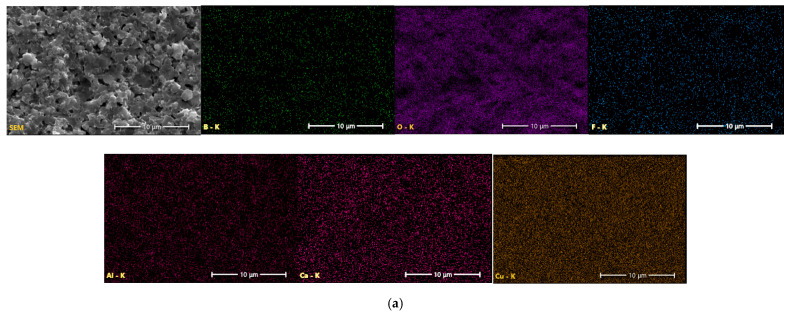

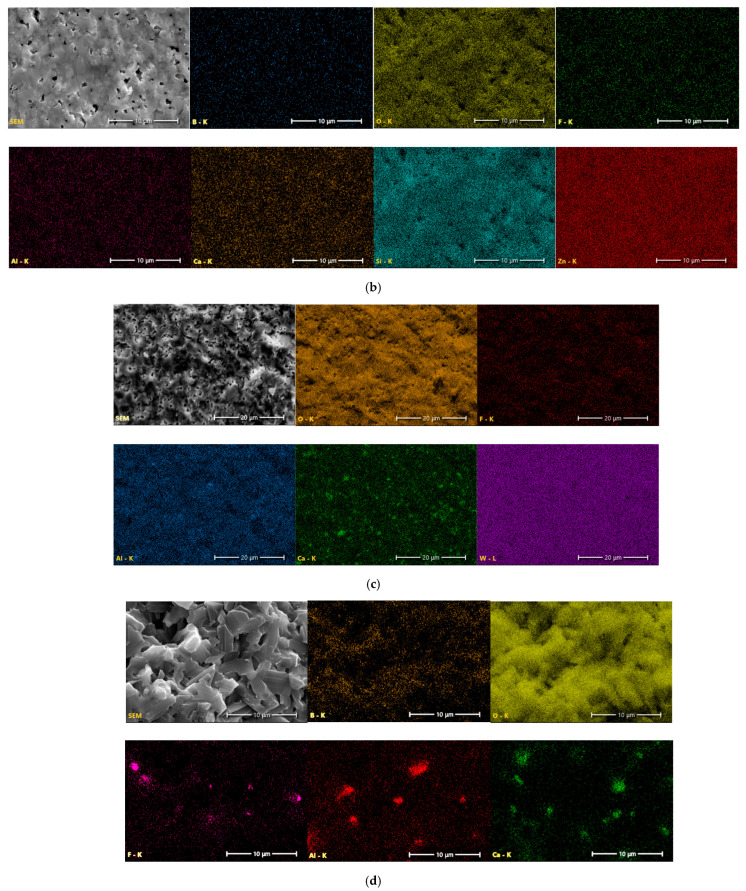
EDS maps of fractured cross-sections: (**a**) CuB_2_O_4_; (**b**) Zn_2_SiO_4_; (**c**) LiBO_2_; (**d**) Li_2_WO_4_ ceramics doped with AlF_3_-CaB_4_O_7_.

Figure 4a, Figure 4b, Figure 4c, and Figure 4d show the comparison of the EDS maps representing the distribution of the constituent elements over the fractured surfaces of doped CuB_2_O_4_, Zn_2_SiO_4_, LiBO_2_, and Li_2_WO_4_ ceramics, respectively. The EDS analysis indicates that the distribution of the main elements is uniform—Cu, B, and O for CuB_2_O_4_ (Figure 4a); Zn, Si, and O for Zn_2_SiO_4_ (Figure 4b); B and O for LiBO_2_ (due to low atomic numbers Li is not detectable and the concentration of B is underestimated in the EDS method) (Figure 4c); and W and O for Li_2_WO_4_ (Figure 4d). Generally, the elements present only in the dopant—Al, F, and Ca—are also uniformly distributed over the sample surfaces. However, Ca exhibits a tendency to form small regions with a higher concentration of LiBO_2_ (Figure 4c) and Li_2_WO_4_ (Figure 4d) ceramics.

**Figure 5:**
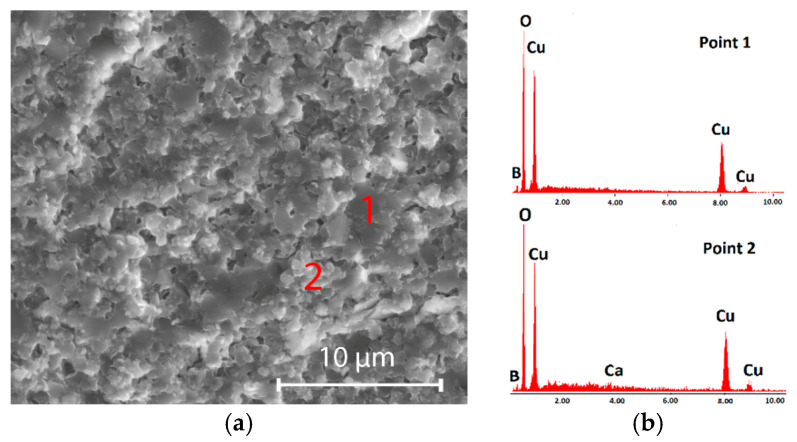
(**a**) SEM image; (**b**) EDS analysis (point 1 and point 2 indicated in Figure 5a) of CuB_2_O_4_ ceramic with 5% AlF_3_-CaB_4_O_7_.

Furthermore, this effect was confirmed for CuB_2_O_4_ ceramics by the EDS point analysis presented in Figure 5b for the points indicated on the SEM image in Figure 5a. It demonstrates a uniform distribution of the main component elements Cu, O, and B. A small enrichment in Ca, being the constituent element of the dopant, was observed at spot 2, which covers the area with more grain boundaries. The results of surface and point EDS analysis (Figure 4 and Figure 5) imply that Ca preferably contributes to the formation of an amorphous phase at grain boundaries. There is a low probability that the Ca^2+^ ion with the ionic radius of 1.00 Å (for coordination number CN = 4) can substitute much smaller ions in the crystal lattice of CuB_2_O_4_, Zn_2_SiO_4_, LiBO_2_, or Li_2_WO_4_ ceramics–Cu^2+^ (0.57 Å, CN = 4), Zn^2+^ (0.60 Å, CN = 4), and Li^+^ (0.59 Å, CN = 4). The small Al^3+^ cation, with an ionic radius of 0.39 Å (CN = 4), can more easily enter the host crystal lattice of the investigated materials. According to the studies of Yang et al., the F^−^ anion, which has a lower ionic radius (1.33 Å) than O^2−^ (1.40 Å), can substitute for oxygen in the crystal lattice or partly fill oxygen vacancies [14].

**Figure 6:**
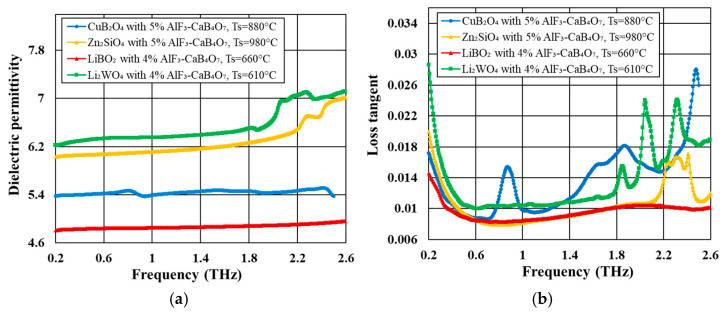
Comparison of the frequency dependences of: (**a**) dielectric permittivity; (**b**) loss tangent for ceramics based on CuB_2_O_4_, Zn_2_SiO_4_, LiBO_2_, and Li_2_WO_4_—all doped with AlF_3_-CaB_4_O_7_.

Figure 6 shows the comparison of dielectric permittivity and dielectric loss as a function of frequency in the range of 0.2–2.6 THz for the doped ceramics. All investigated materials exhibit low and stable dielectric permittivity (4.8–6.5) and relatively small loss tangent (0.008–0.015) in a broad frequency range of 0.2–1.4 THz. The most advantageous dielectric properties are demonstrated by doped LiBO_2_—a very low dielectric permittivity of 4.8 and a low loss tangent of 0.008 at 1 THz.

**Figure 7:**
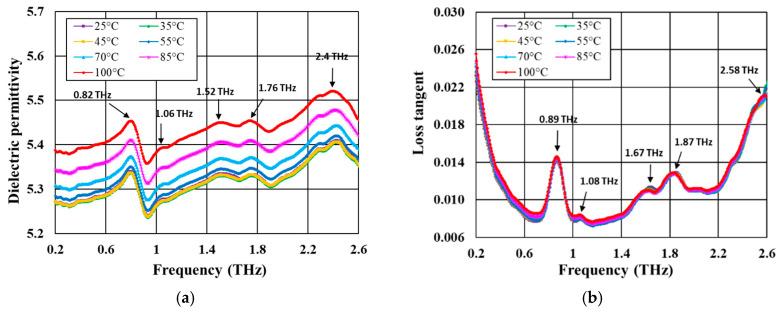
Frequency dependence in the 0.2–2.6 THz range of: (**a**) dielectric permittivity; (**b**) loss tangent for ceramics based on CuB_2_O_4_ doped with AlF_3_-CaB_4_O_7_.

Figure 7 shows the frequency dependence of dielectric permittivity and loss tangent for doped CuB_2_O_4_-based ceramic, measured at various temperatures in the range of 25–100 °C. In the dielectric permittivity-frequency plots, a few small characteristic peaks occur in the investigated frequency range related to phonon modes—at 0.82, 1.06, 1.52, 1.76, and 2.4 THz. The corresponding peaks in the loss tangent-frequency plots are slightly shifted toward higher frequencies. The temperature that increases in the investigated range of 25–100 °C causes a slight increase in dielectric permittivity up to 55 °C and a more pronounced increase at higher temperatures. The position of the peaks is weakly dependent on temperature, although a small shift to lower frequencies can be observed with increasing temperature.

The calculated values of the temperature coefficient of resonant frequency in the temperature range 20–55 °C at 1 THz are −24 ppm/°C for doped Zn_2_SiO_4_, −106 ppm/°C for doped CuB_2_O_4_, −133 ppm/°C for doped LiBO_2,_ and −160 ppm/°C for doped Li_2_WO_4_. The τ_f_ values in the temperature range 20–55 °C at 1 THz determined previously by the authors for undoped ceramics are −5 ppm/°C for CuB_2_O_4_ [13], −5 ppm/°C for Zn_2_SiO_4_ [23], −253 ppm/°C for LiBO_2_ [32], and −164 ppm/°C for Li_2_WO_4_ [35].

The investigated ceramics have been the subject of several studies using Raman and FTIR spectroscopy, which provided basic knowledge about their vibrational spectra, molecular composition and structure, and chemical bonding, including rotations and oscillations of the structural units—CuO_4_, ZnO_4_, SiO_4_, LiO_4_, and WO_4_ tetrahedra, as well as planar triangles BO_3_ [25,26,27,37,38]. These studies comprised a frequency range much higher (400–4000 cm^−1^) than that investigated in the present study (0.3–100 cm^−1^). The phonon modes in the 0.1–3 THz frequency range examined in the present research were rarely analyzed [39,40]. For willemite Zn_2_SiO_4_, a strong absorption peak found around 2.3 THz was assigned to a low-frequency phonon mode by Nedelcu et al. [39]. The copper borate CuB_2_O_4_ hosts a relatively narrow-phonon mode at approximately 0.92 THz, as reported by Białek et al. [40].

The applied additive AlF_3_-CaB_4_O_7_ is composed of ions with small ionic polarizabilities: Al^3+^ (0.53 Å^3^), F^−^ (0.82 Å^3^), Ca^2+^ (1.79 Å^3^), B^3+^ (0.12 Å^3^), and O^2−^ (1.79 Å^3^), which should be advantageous for lowering ionic polarization in the doped materials. From Table 2, it can be seen that the applied dopant does not deteriorate the good dielectric properties of the undoped ceramics at terahertz frequencies. The values of dielectric permittivity and dielectric loss for three doped ceramics are close to those of undoped ceramics. For doped LiBO_2_ ceramics, the additional advantageous effects of restricted reactivity with water, diminishing of the temperature coefficient of resonant frequency, and suppression of dielectric loss are attained. The less beneficial impact of the applied dopant was noticed only for Li_2_WO_4_, related to an increase in dielectric permittivity. This can presumably be caused by filling the pores with an amorphous phase and better pore elimination due to doping, which results in an enhanced dielectric permittivity, although elucidation of this effect can become an object of a separate study and is left for future investigations.

Low dielectric permittivity of ceramic materials is determined by several intrinsic factors related to their composition and crystal structure—low ionic polarizability, a high contribution of covalent bonds, high packing fraction, low average bond length, and cell volume [41,42,43]. Generally, the dielectric losses originate from both intrinsic sources related to the crystal structure and extrinsic sources related to processing. In the terahertz frequency range, intrinsic dielectric losses resulting from phonon vibrations in the crystal lattice are dominant. According to the damped harmonic oscillator model, the imaginary part of dielectric permittivity (and accordingly loss tangent) increases proportionally with frequency in the broad range (GHz to THz) corresponding to the microwave to submillimeter wavelength [44], which was experimentally confirmed for several LTCC materials [23,24,28,45]. However, extrinsic sources of losses related to pores, secondary crystalline and amorphous phases, grain boundaries, defect ordering, and microstructural defects cannot be fully excluded in the real, imperfectly sintered ceramics.

**Figure 8:**
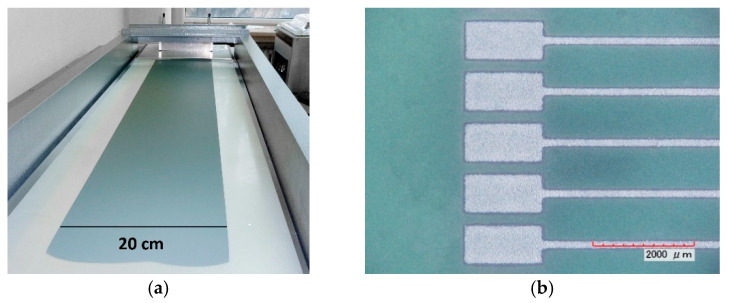
(**a**) LTCC green tape based on CuB_2_O_4_; (**b**) Test conductor pattern screen printed on CuB_2_O_4_ green (unfired) sheet.

The developed ceramics were successfully used for the preparation of slurries and subsequent casting of defect-free green tapes, which have good smoothness, flexibility, and mechanical strength. Figure 8a shows a green (unfired) tape based on doped CuB_2_O_4_. Besides a properly adjusted low sintering temperature, feasibility in ULTCC/LTCC technology requires compatibility with conductor layers screen printed and cofired or postfired on the top of the substrates or embedded inside the multilayer structure.

Minimizing the electrical contact resistance between the conductive layer and the dielectric substrate is important for achieving the desired performance of electronic circuits. This resistance is influenced by the surface roughness of the substrate, the adhesion of thick film conductors, and interfacial reactions between metallic and ceramic layers. Ensuring chemical compatibility between the conductor film and substrate enables avoiding interfacial reactions, while a proper surface finish and sintering profile entail good contact and minimize voids and air gaps at the interface.

**Figure 9:**
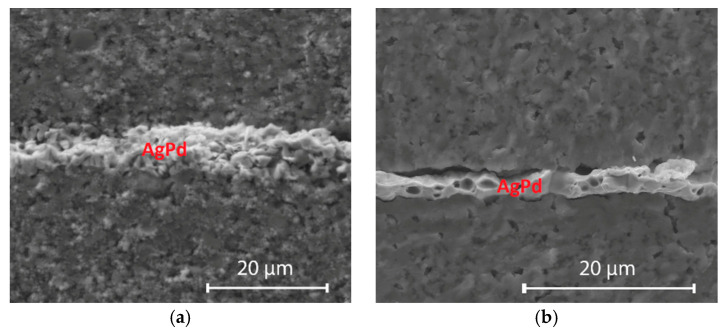

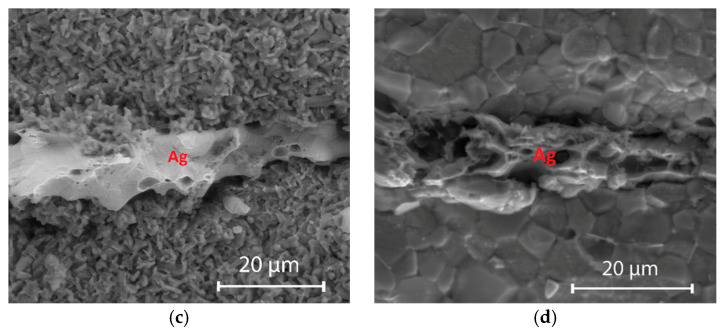
SEM images of metallic layer-ceramic layer interfaces in LTCC/ULTCC structures: (**a**) doped CuB_2_O_4_; (**b**) doped Zn_2_SiO_4_; (**c**) doped LiBO_2_; (**d**) doped Li_2_WO_4_.

The fabricated green tapes show good compatibility with the applied commercial thick film conductor pastes based on Ag and AgPd. For the materials used in this work, the lack of reactivity between Ag or AgPd and ceramics has been proven previously [13,22,31,34]. Figure 6b illustrates a test Ag pattern screen printed on an unfired CuB_2_O_4_ sheet. It was demonstrated that it is possible to obtain high-quality thick film conductor paths with a width of about 100 μm, sharp edges, and compatibility with the developed green tapes. The resolution of test conductor patterns after screen printing and firing processes is acceptable from the point of view of future applications of ceramic materials, e.g., as substrates for microwave antennas. Good compatibility with commercial Ag and/or AgPd thick film pastes was found for both the undoped and doped with AlF_3_-CaB_4_O_7_ ceramics under investigation. Figure 9 illustrates good cooperation of internal metallic thick films (Ag paste for LiBO_2_ and Li_2_WO_4_, AgPd paste for CuB_2_O_4_ and Zn_2_SiO_4_) with cofired ceramic layers. The potential applicability of the AlF_3_–CaB_4_O_7_ additive to other LTCC-relevant ceramic systems (e.g., gallates, phosphates, and molybdates) appears to be a viable direction for further investigation.

Two of the investigated ceramics doped with AlF_3_-CaB_4_O_7_, Zn_2_SiO_4_, and CuB_2_O_4_, with the sintering temperatures of 900–980 °C, are feasible in LTCC technology, while the other two doped materials with the ultra-low sintering temperatures of 600–660 °C, LiBO_2_, and Li_2_WO_4_, can be fabricated in ULTCC technology.

## 4. Conclusions

It was demonstrated that the additive, which consists of AlF_3_ mixed with CaB_4_O_7_ in a 1:1 molar ratio, can be successfully used to decrease sintering temperatures and enhance feasibility in LTCC/ULTCC technology of four low dielectric permittivity materials, based on CuB_2_O_4_, Zn_2_SiO_4_, LiBO_2_, and Li_2_WO_4_, without compromising on their excellent dielectric properties in the terahertz frequency range and compatibility with cheap commercial conductor pastes.

The fabricated ceramics exhibit very good dielectric properties at terahertz frequencies—a low and stable dielectric permittivity (4.8–6.5) and a small loss tangent (0.008–0.015) in a broad range of 0.2–1.4 THz. The applied dopant was found to be the most effective for the improvement of the dielectric properties of LiBO_2_ ceramics. The developed materials can be applied as substrates for 5G/6G communication systems.

## Figures and Tables

**Table 1:** Comparison of characteristic temperatures for undoped and AlF_3_-CaB_4_O_7_-doped ceramics based on CuB_2_O_4_, Zn_2_SiO_4_, LiBO_2_, and Li_2_WO_4_.

Ceramics	Melting Temperature, °C	Sintering Range, °C	Start of Shrinking, °C
Undoped CuB_2_O_4_	1002	950–980	891
Doped CuB_2_O_4_	990	900–940	822
Undoped Zn_2_SiO_4_	1470	1320–1350	1180
Doped Zn_2_SiO_4_	1373	950–980	896
Undoped LiBO_2_	819	670–700	630
Doped LiBO_2_	810	650–680	616
Undoped Li_2_WO_4_	737	640–650	576
Doped Li_2_WO_4_	714	590–620	561

**Table 2:** Comparison of sintering temperatures and dielectric permittivities for undoped and doped CuB_2_O_4_, Zn_2_SiO_4_, LiBO_2_, and Li_2_WO_4_ ceramics.

Ceramics	Dielectric Permittivity at 1 THz		Sintering Temperature, °C	
	Doped	Undoped	Doped	Undoped
CuB_2_O_4_	5.4	5.5	900	960
Zn_2_SiO_4_	6.1	6.0	960	1320
Li_2_WO_4_	6.3	5.1	600	640
LiBO_2_	4.8	4.8	660	700

## Data Availability

The original contributions presented in this study are included in the article, and further inquiries can be directed to the corresponding author.

## Abbreviations

The following abbreviations are used in this manuscript:

LTCC	Low-temperature co-fired ceramics
ULTCC	Ultra-low temperature co-fired ceramics
SEM	Scanning electron microscope
EDS	Energy dispersive spectroscopy
XRD	X-ray diffraction
TDS	Time domain spectroscopy
